# Supplementary material for: Mitochondrial ATP synthesis is essential for efficient gametogenesis in Plasmodium falciparum
Source: Commun Biol. 2024 Nov 16;7:1525. doi: 10.1038/s42003-024-07240-z (PMC11569237; doi:10.1038/s42003-024-07240-z)
Supplement: Supplementary file 1 — Supplementary Information [file 42003_2024_7240_MOESM1_ESM.pdf]

Supplementary Figure 1 – Labelling gametocytes with male and female gametocyte-specific antibodies.

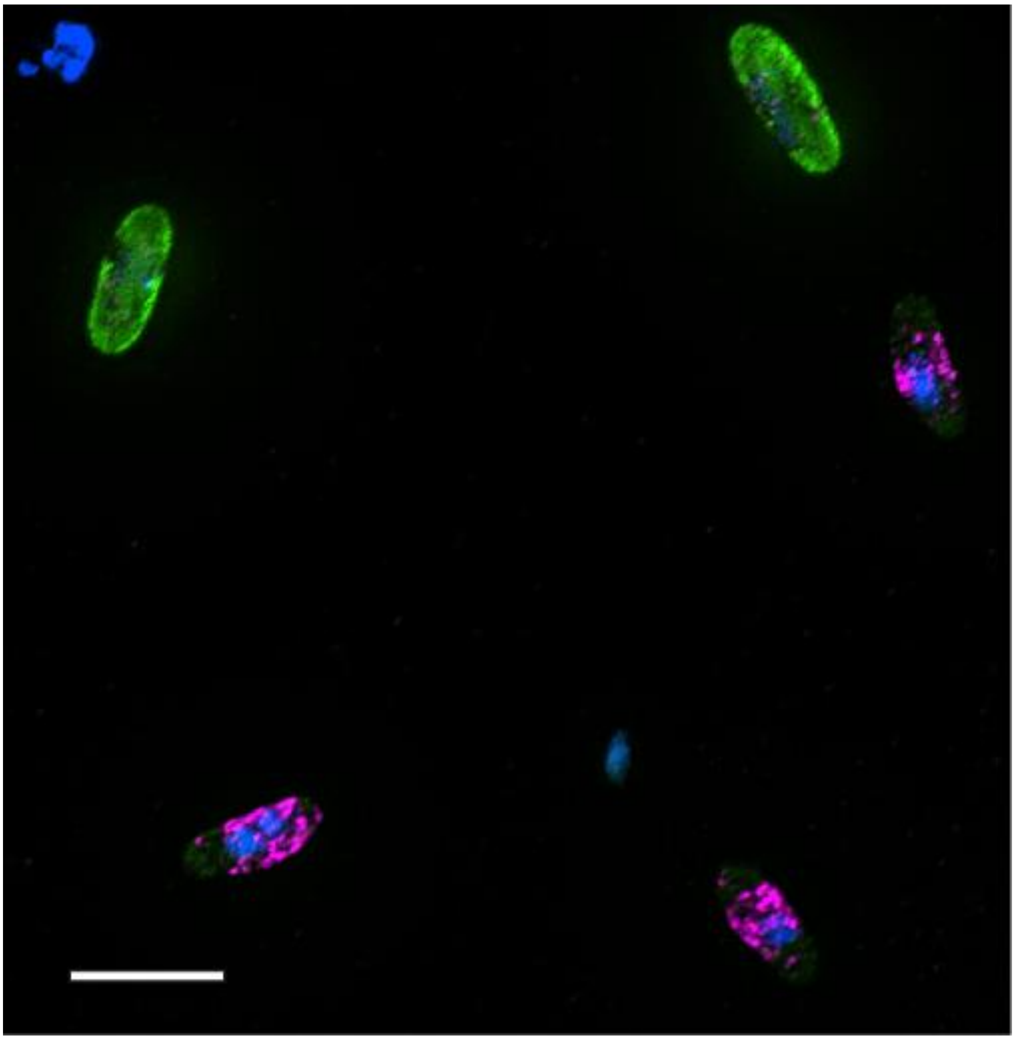

Male and female Stage V gametocytes stained with the putative male gametocyte marker anti-LDH2 (green), the established female marker anti-PfG377 (magenta) and DAPI (blue). Scale bar = 12 µm.

Supplementary Figure 2 – Specificity of the anti-LDH2 and anti-PfG377 polyclonal antibodies.

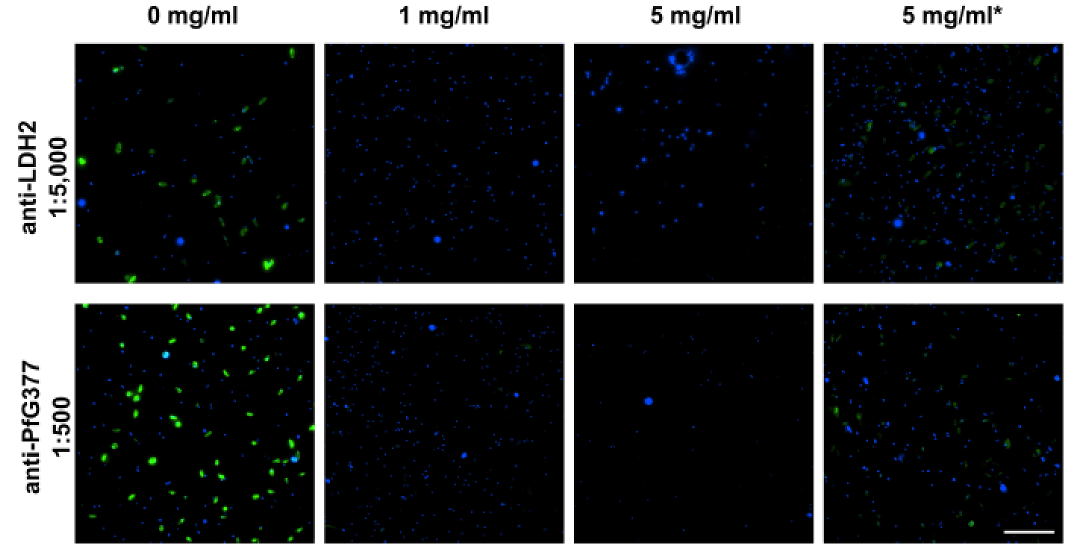

Antibody (green); DAPI (blue). Antibodies were incubated for 2 h with the specified concentration of the two peptides used to generate each antibody and then used to stain gametocytes. “5 mg/ml*” indicates blocking the antibody with the peptides used to immunise against the other protein. This high concentration showed reduced, but specific staining. Scale bar = 35 µm.

Supplementary Figure 3 – Extended version of Figure 2A showing a higher saturation image of the DAPI staining.

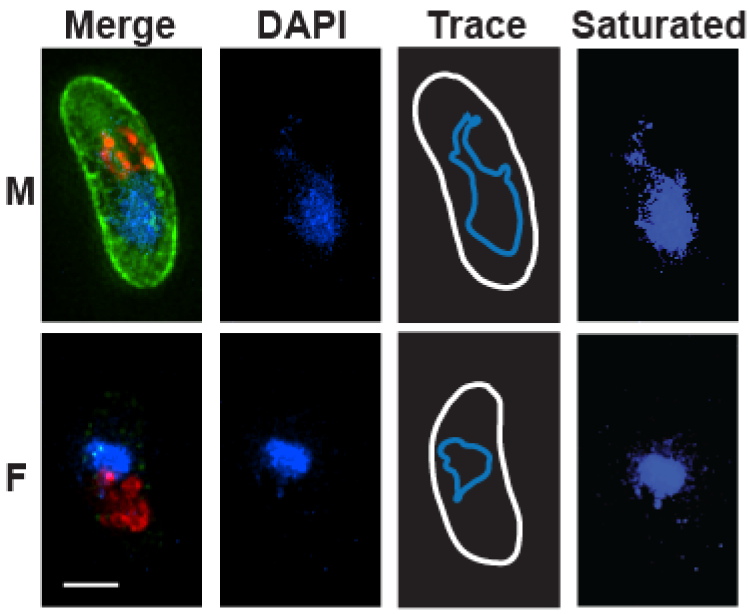

All images used for analysis in this manuscript were captured and analysed as 16-bit images containing 0-65,536 shades of grey. Hence some fainter detail is lost when reproducing images in the manuscript figures at 8-bit (0-255 shades of grey). Scale bar = 3 µM.

Supplementary Figure 4 – Quantification of MitoTracker staining in male and female gametocytes.

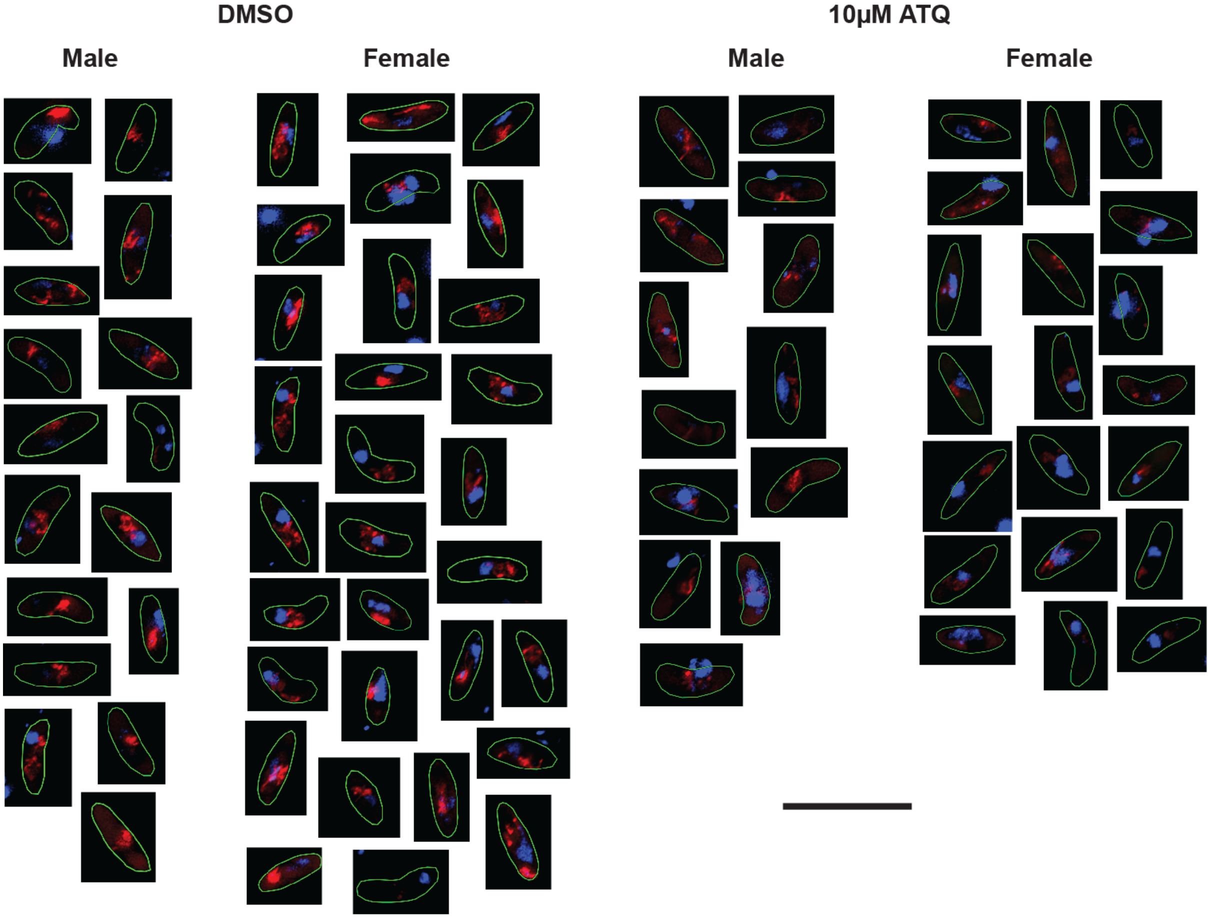

A montage of individual male and female gametocytes from the analysis in Figure 2 and Figure 3. Shown here are the outline of the cell (green), MitoTracker staining (red) and DAPI staining (blue). Mitochondrial morphology varies substantially between cells, with no clear pattern distinguishing male and female gametocytes. Gametocytes treated with 10μM atovaquone (ATQ) show reduced MitoTracker incorporation. Scale bar (black) = 16 μM.

Supplementary Figure 5 – Titrating MitoTracker labelling of gametocytes

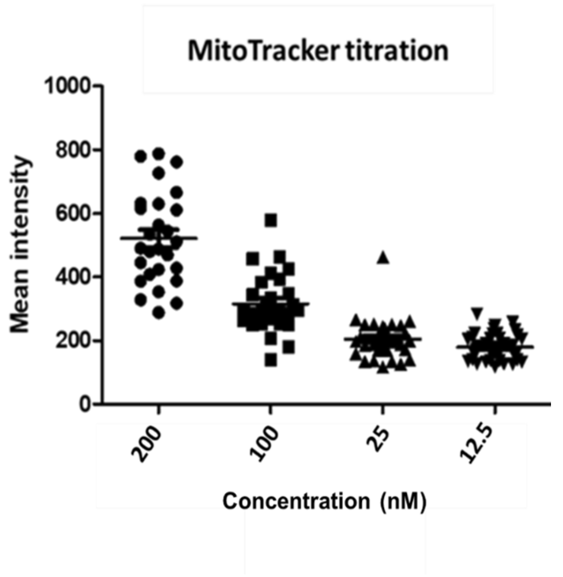

Live gametocytes were treated with decreasing concentrations of MitoTracker for 25 minutes before being washed and fixed. Intensity of MitoTracker staining was then calculated for individual cells by fluorescence imaging and quantitative image analysis. A detectable signal was observed in gametocyte mitochondria treated with as little as 12.5nM MitoTracker. Black bar indicates the mean.

Supplementary Figure 6 – Short term incubation in glucose-free RPMI has no effect on male gametogenesis.

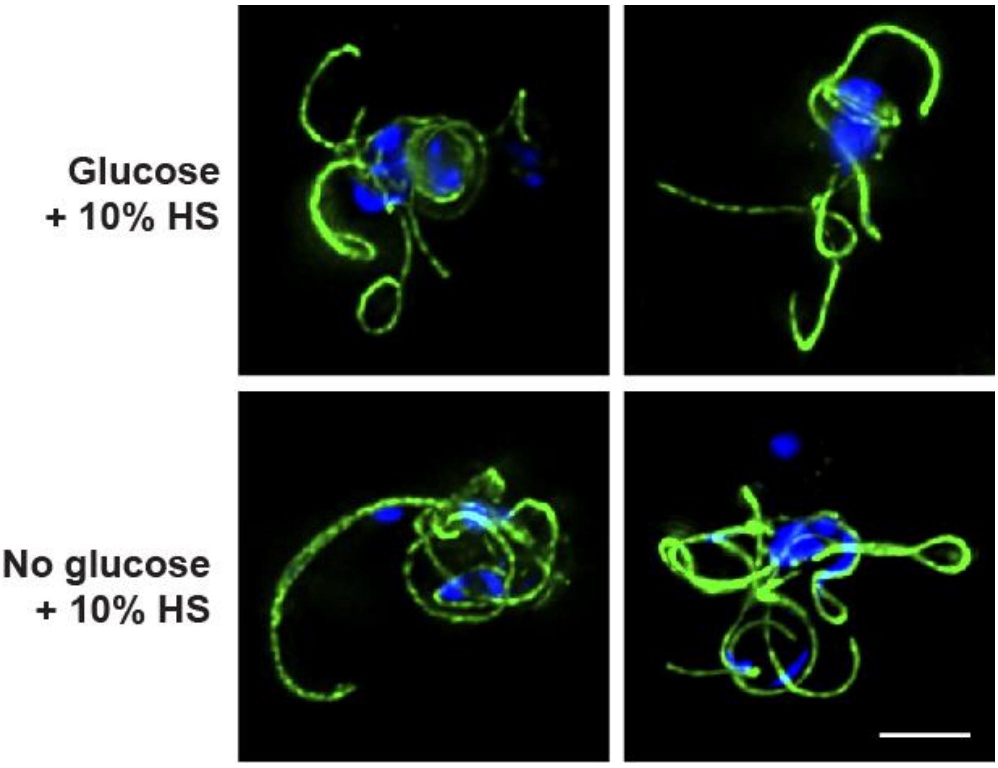

Gametocytes were incubated for 1 h with culture medium containing RPMI with or without glucose, plus 10% human serum (HS). They were then triggered in condition-matched ookinete medium, fixed 20 min later, and stained with anti-alpha tubulin II (green) and DAPI (blue). We conclude that 10% human serum contains sufficient endogenous glucose to fuel exflagellation. Scale bar = 5 µM.
