# Supplementary material for: Mitochondrial ATP synthesis is essential for efficient gametogenesis in Plasmodium falciparum
Source: Commun Biol. 2024 Nov 16;7:1525. doi: 10.1038/s42003-024-07240-z (PMC11569237; doi:10.1038/s42003-024-07240-z)
Supplement: Supplementary file 2 — Description of Additional Supplementary Materials [file 42003_2024_7240_MOESM2_ESM.pdf]

Description of Additional Supplementary Files

File name: Supplementary Data 1

Description: All data supporting the findings of this study
